# Influence of In Vitro Human Digestion Simulation on the Phenolics Contents and Biological Activities of the Aqueous Extracts from Turkish *Cistus* Species

**DOI:** 10.3390/molecules26175322

**Published:** 2021-09-01

**Authors:** Yiğit İnan, Selin Akyüz, Inci Kurt-Celep, Engin Celep, Erdem Yesilada

**Affiliations:** Department of Pharmacognosy, Faculty of Pharmacy, Yeditepe University, Atasehir, 34755 Istanbul, Turkey; yigit.inan@yeditepe.edu.tr (Y.İ.); selin.akyuz@yeditepe.edu.tr (S.A.); incikurt00@gmail.com (I.K.-C.); ecelep@yeditepe.edu.tr (E.C.)

**Keywords:** Turkish *Cistus* species, antioxidant activity, human digestion simulation, HPTLC, diabetes

## Abstract

Oxidative stress is one of the significant precursors of various metabolic diseases such as diabetes, Parkinson’s disease, cardiovascular diseases, cancer, etc. Various scientific reports have indicated that secondary plant metabolites play an important role in preventing oxidative stress and its harmful effects. In this respect, this study was planned to investigate the phenolic profile and antioxidant and antidiabetic potentials of the aqueous extracts from Turkish *Cistus* species by employing in vitro methods. In vitro digestion simulation procedure was applied to all extracts to estimate the bioavailability of their phenolic contents. Total phenolic, flavonoid, phenolic acid and proanthocyanidin contents were determined for all phases of digestion. In addition, changes in the quantity of the assigned marker flavonoids (tiliroside, hyperoside and quercitrin) were monitored by High-Performance Thin Layer Chromatography (HPTLC) analysis. The antioxidant activity potentials of the extracts were studied by various methods to reveal their detailed activity profiles. On the other hand, in vitro α-amylase and α-glucosidase enzymes and advanced-glycation end product (AGE) inhibitory activities of the extracts were determined to evaluate the antidiabetic potentials of extracts. The results showed that aqueous extracts obtained from the aerial parts of Turkish *Cistus* species have rich phenolic contents and potential antioxidant and antidiabetic activities; however, their bioactivity profiles and marker flavonoid concentrations might significantly be affected by human digestion. The results exhibited that total phenolic contents, antioxidant activities and diabetes-related enzyme inhibitions of the bioavailable samples were lower than non-digested samples in all extracts.

## 1. Introduction

The Cistaceae family is composed of shrubs, annual or perennial herbaceous plants, and the genus *Cistus* is one of the broadly distributed members of this family. More than 50 *Cistus* species are distributed worldwide, and they are commonly called “rockrose” [1]. Previous in vitro and in vivo investigations demonstrated that *Cistus* species possess antiviral, antidiabetic, antioxidant, antimicrobial and anti-inflammatory activities [2,3]. Different phenolic compounds (phenolic acids, flavonoids, proanthocyanidins) and terpenes were isolated from *Cistus* species, and their therapeutic benefits are generally attributed to these components [4,5].

In Turkey, five *Cistus* species grow naturally, i.e., *C. salviifolius* L., *C. parviflorus* Lam., *C. monspeliensis* L., *C. laurifolius* L. and *C. creticus* L. [6]. In the ethnobotanical records of Turkish folk medicine, various organs of *Cistus* species are frequently documented as a remedy. Infusions prepared from the branches of *C. laurifolius, C. salviifolius* and *C. creticus* are ingested orally against diabetes in Edremit (Balıkesir) district [7]. Decoctions prepared from the flowers of *C. creticus* and *C. salviifolius* are used internally against peptic ulcer in Marmaris (Muğla) [8], while a decoction of the unopened flower buds of *C. laurifolius* is used for the same purpose. In Western Anatolia, the decoction of *C. laurifolius* leaves is used internally against fever and stomachache and externally, via bathing, against rheumatic pain [9].

It is a well-proven fact that the elevated aggregation of reactive oxygen species (ROS) triggers oxidative stress, which is one of the significant precursors of various metabolic disorders such as cancer, diabetes, cardiovascular problems, Alzheimer’s disease, etc. [10]. Therefore, antioxidant utilization has become a common holistic approach for preventing or treating such conditions in current scientific practice. The antioxidant activities of the plant extracts have been reported by a tremendous number of researchers [11,12,13,14]. As a common approach, the antioxidant potential of the plant extracts is generally devoted to their phenolic contents. A large body of evidence is available in the scientific literature that *Cistus* species are also rich in phenolic profiles and eventually have a considerable degree of antioxidant activity. However, the bioavailability concept of these phytochemicals in the body has not been considered in most of these studies.

It is a well-known fact that gastrointestinal tract conditions influence phenolic compounds due to different pH conditions, enzyme actions and microbiota. On the other hand, the chemical structures of phenolic compounds and the plant matrix are also important factors affecting their bioavailability [15]. Therefore, in the present investigation, the in vitro digestion simulation method was applied to all extracts to estimate the bioavailability of phenolic contents. In order to monitor the transitions, total phenolic, flavonoid, phenolic acid and proanthocyanidin contents were determined in all phases of digestion. Moreover, the antioxidant activities of the extracts were studied by mechanistically different spectrophotometric methods to reveal their comprehensive activity profiles. The antioxidant potentials of all samples obtained by the digestion process were investigated with DPPH and DMPD (free radical scavenging), FRAP and CUPRAC (metal-reducing potential) and TOAC (total antioxidant capacity) assays. Previously, tiliroside, hyperoside and quercitrin were determined as the marker flavonoids of *Cistus* species by Guzelmeric et al. [16]. Therefore, the qualitative and quantitative determination of these flavonol glycosides was carried out with the High-Performance Thin Layer Chromatography system and their bioavailability indexes were estimated.

Diabetes mellitus (DM) is a common metabolic disorder and is described by decreased insulin secretion by pancreatic β-cells or lack of responses of the body to insulin. There are two types of DM: Insulin dependent (Type I) and non-insulin dependent (Type II) [17]. One of the treatment strategies of Type II DM is to control postprandial hyperglycemia, which is defined as “a significant rise of blood sugar concentration in the bloodstream following a meal”. Inhibition of the key digestive enzymes, including α-amylase and α-glucosidase, is essential to controling postprandial hyperglycemia. In the gastrointestinal system, α-amylase digests the starch into reducing sugars such as amylodextrin, lactose and maltose, and α-glucosidase breaks these sugars into glucose. Therefore, inhibition of digestive enzymes is regarded as a possible mode of action to treat postprandial hyperglycemia [18]. On the other hand, elevated blood glucose levels may trigger the formation of AGEs which are defined as “compounds formed as a result of enzymatic glycation reaction (Maillard) between reducing sugars and proteins, nucleic acids and lipids”. Elevated accumulation of AGEs in the body may induce many diabetic complications, including nephropathy, neuropathy, retinopathy, etc. [19]. Aminoguanidine, pimagedine and metformin are examples of synthetic inhibitors for AGEs and acarbose, miglitol and voglibose are synthetic inhibitors for digestive enzymes and have been in use for the past decades [20,21]. However, clinical trials and in vivo experiments demonstrated the side effects of these synthetic inhibitors, such as hepatotoxicity, abdominal distention, flatulence, meteorism, anaemia, vomiting, heart failure, etc. [21,22]. Due to such harmful effects, multiple studies have involved the inhibitory potentials of the plant extracts on AGEs [23,24,25]. It has been reported that phytochemicals, particularly phenolic compounds such as phenolic acids, flavonoids and proanthocyanidins, significantly inhibited the formation of AGEs and related enzyme actions, i.e., α-amylase and α-glucosidase [26,27,28].

Since water extraction (infusion or decoction) is the common preparation technique in traditional medicine, this study was carried out on the aqueous extracts from Turkish *Cistus* species before and after the in vitro gastrointestinal digestion simulation. In this respect, the phenolic profiles and antioxidant and antidiabetic potentials of the aqueous extracts and their digestion metabolites were comparatively investigated. According to the reference survey, inhibitory activities of *Cistus* extracts on AGEs were studied for the first time in this study. In addition, quantitative analysis of the marker flavonoids was also accomplished by HPTLC analysis. In vitro digestion simulation technique was applied to all extracts to monitor the alterations in the concentrations and the biological activity profiles of the phenolic compounds in the gastrointestinal conditions.

## 2. Results

### 2.1. Estimation of the Phenolic Contents of the Samples

According to the results shown in Table 1, aqueous extract of *C. salviifolius* had higher total flavonoid, phenolic and phenolic acid contents than other studied species, while ND (non-digested) samples of *C. creticus* and *C. laurifolius* possessed the highest proanthocyanidin contents. The most significant decrease was detected in the total proanthocyanidins contents of all extracts. Proanthocyanidin amounts of IN (bioavailable) samples were undetectable in all aqueous extracts. As a result, the phenolic contents of the aqueous extracts were negatively affected by the in vitro human digestion simulation procedure.

**Figure 1:**
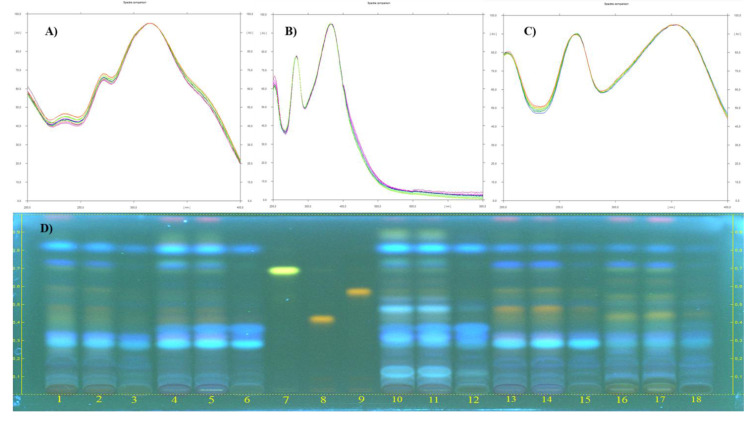
(**A**) Overlay UV spectra of tiliroside and the corresponding spots in the tracks of all extracts. (**B**) Overlay UV spectra of hyperoside and the corresponding spots in the tracks of all extracts. (**C**) Overlay UV spectra of quercitrin and the corresponding spots in the tracks of all extracts. (**D**) HPTLC chromatograms of: 1. CCA ND, 2. CCA PG, 3. CCA IN, 4. CLA ND, 5. CLA PG, 6. CLA IN, 7. Tiliroside (R*f* ≈ 0.65), 8. Hyperoside (R*f* ≈ 0.35), 9. Quercitrin (R*f* ≈ 0.45), 10. CMA ND, 11. CMA PG, 12. CMA IN, 13. CPA ND, 14. CPA PG, 15. CPA IN, 16. CSA ND, 17. CSA PG, 18. CSA IN. Mobile phase: EtOAc/CHCl_2_/CH_3_COOH/HCOOH/H_2_O (100:25:10:10:11); Derivatization: NPR reagent. Visualization: 366 nm.

As presented in Table 2, tiliroside and hyperoside contents in the aqueous extract of *C. salviifolius* were relatively higher than those of the other species, while quercitrin was not found. On the other hand, quercitrin was found in the highest concentration in all simulation samples of the aqueous extract from *C. creticus*, but its concentration reduced significantly in bioavailable samples. Additionally, HPTLC chromatogram and overlay UV spectras of references and the corresponding spots in the tracks of all extracts were presented in Figure 1.

### 2.2. Estimation of Antioxidant Activity of the Samples

As presented in Table 3, bioavailable samples of *Cistus* extracts exhibited weaker radical scavenging antioxidant activity than their non-digested and post-gastric counterparts. ND and PG samples of all aqueous extracts showed significant DPPH radical scavenging activity and possessed lower EC_50_ values than reference compound BHT (EC_50_ value: 5.83 ± 0.2 μg/mL). However, all extracts displayed a weaker DMPD radical scavenging activity than reference compound Trolox (5.82 ± 0.37 μg/mL). ND, PG and IN samples of CPA possessed better DMPD activity compared to samples of other extracts. 

Similar to radical scavenging activity assays, bioavailable samples of *Cistus* extracts also showed weaker metal-reducing and total antioxidant activities than the non-digested and post-gastric samples. All ND samples of the extracts displayed significant ferric reducing antioxidant activity, which was stronger than the reference compound BHT (4.06 ± 0.42 mM FeSO4 equivalent). Among PG samples, only CSA (4.44 ± 0.16 mM FeSO4 equivalent) had better activity than BHT. In CUPRAC assay, ND and PG samples of CSA were detected as most potent among the samples of other species. Discretely, IN sample of CCA had better CUPRAC activity than bioavailable samples of other aqueous extracts. 

### 2.3. Diabetes-Related Enzyme Inhibition Activity

As indicated in Table 4, concentration-dependent enzyme inhibitory activity was seen in all aqueous extracts. While ND samples of CPA and CSA (75.89% ± 0.62, 80.34% ± 0.19, respectively) exhibited somewhat higher α-amylase inhibitory activity than acarbose (75.80% ± 0.02) at the 1 mg/mL concentration; only the ND sample of CSA exhibited higher α-glucosidase inhibitory activity than the reference compound quercetin in both concentrations.

To sum up, the aqueous extract of *C. salviifolius* exhibited a better digestive enzyme inhibitory activity than the extracts of other species. Additionally, IN samples of all aqueous extracts revealed lower enzyme inhibitory activities comparing to ND samples.

### 2.4. AGEs Inhibitory Activity

As presented in Table 4, concentration-dependent AGE inhibitory activity was observed in all aqueous extracts. ND samples of CCA, CPA and CSA exhibited better inhibitory activity than the reference compound quercetin in both 0.5 and 1 mg/mL concentrations. However, only *C. salviifolius* extract displayed better inhibitory activity than quercetin among IN samples of the extracts. Bioavailable samples of aqueous extracts showed lower AGE inhibitory activities compared to non-digested samples. According to the results, the ND sample of aqueous extract of *C. salviifolius* possessed the highest AGE inhibitory activity. However, the IN sample of *C. monspeliensis* aqueous extract displayed the weakest AGE inhibition potential in tested concentrations.

## 3. Discussion

Free radicals might attack proteins, lipids, or DNA to procure an electron, resulting in various health problems. For this reason, it is essential to balance the antioxidant system of the body and the free radicals formed by oxidation. In case of the deterioration of this balance, oxidative stress arises [29]. Oxidative stress is one of the main factors resulting in various chronic disorders such as cancer, diabetes, cardiovascular diseases, Alzheimer’s disease, etc. While there are self-antioxidant systems in the human body to combat the oxidative damage to tissues and organs, these defence systems may lose their efficiency due to different conditions such as alcohol over-consumption, smoking, stress, chronic drug usage, radiation, etc. [30]. Various scientific reports have indicated that secondary plant metabolites play a significant role in preventing oxidative stress and its harmful effects [13,14,31]. Even though bioavailability is an important parameter affecting the bioactivity of these compounds, it has not been taken into account in most studies. However, it is well known that the secondary metabolites are subjected to structural transformations due to different pH conditions, enzymatic activity and body temperature in the gastrointestinal system. In addition, the chemical characteristics of the secondary metabolites such as molecular weight, polarity and degree of binding to macromolecules in the plant matrix are also significant factors affecting their bioavailability [13]. Accordingly, absorption of the compounds or their metabolites to the bloodstream once ingested is essential in order to exert their systemic physiological effects. In vitro digestion simulation models may provide evidence for the assessment of the possible bioavailability characteristics of substances. While confirmation in human trials is necessary to claim any functional property, in vitro simulation models are broadly used as alternatives to in vivo studies or human trials, which are often ethically debatable, resource intensive, expensive and time consuming [32].

Several researchers have also investigated the antioxidant potential of extracts prepared from different organs of *Cistus* species. According to the literature survey, this is the first study on *Cistus* species regarding the bioavailability of phenolic substances and their impact on antioxidant activity by employing an in vitro digestion simulation model. Karas et al. [33] suggested that 10% of the polyphenolic components remain undigested in the plant matrix and 90% of them are subjected to digestion in the gastric or intestinal phase (approximately 48% and 52%, respectively). As mentioned earlier, all phenolic amounts in aqueous extracts were negatively affected by the in vitro human digestion simulation procedure. Thus, significant losses were detected in the phenolic contents of the bioavailable samples of all extracts. Moreover, total proanthocyanidin concentrations of IN samples were below the limit of detection in all aqueous extracts. Various studies also prompted the negative influence of digestion procedure on phenolic compounds in the plant extracts and thus related bioactivities. For instance, we reported that the total phenolic content of *Salvia virgata* Jacq. was also adversely affected by the digestion procedure in our previous study. In addition, the amounts of major metabolites of the extract, i.e., rutin and rosmarinic acid, tended to decline [13]. In contrast, several studies have reported contradictory results. In the study by Celep et al. [34], they observed the increment of the total phenolic acid, flavonoid and phenolic amounts in the bioavailable samples of methanolic extract from *Hypericum perfoliatum* L. In fact, the main metabolites, quercitrin, chlorogenic and gallic acid, had bioaccessibility ratios over 100%. These studies showed that the effects of the digestion procedure might vary according to plant materials. In order to clarify these differences, it is essential to consider the mechanism of action of the digestion system on phenolic substances. Serra et al. [35] suggested that phenolic compounds are mainly found in glycosides, polymers and ester forms in the plant matrix and are hydrolysed in the digestive system before absorption. Various factors may influence the structural transformations of the phenolic compounds in the gastrointestinal tract. For instance, compounds with higher molecular weights, such as proanthocyanidins or procyanidins, need to be hydrolysed before absorption in the gut. The structure of the plant matrix is also a prominent factor for the bioavailability of phenolics; phenolic compounds can bind to macromolecules in the plant matrix, such as fibers, proteins and lipid molecules. Thus, only the liberated phenolic components from the matrix may become absorbable from the gastrointestinal tract. Moreover, different pH values and enzymatic actions of the intestinal microbiota are among the other crucial factors affecting the transformation in the chemical structure of phenolic compounds [36]. In light of these data, we can hypothesise that different results obtained from similar types of experimental studies may be due to the complexity of the digestion system and the composition of the plant matrix. As presented in Table 3, the bioavailable samples of *Cistus* extracts exhibited a weaker antioxidant activity than the non-digested and post-gastric counterparts due to their lower phenolic contents.

Moreover, both extracts of *C. salviifolius* displayed better antioxidant activity in DPPH, CUPRAC, FRAP and TOAC assays when compared to other species. Besides, both extracts of *C. parviflorus* showed higher DMPD radical scavenging activity than the extracts of other species. As indicated in Table 1, the total phenolics, flavonoids and phenolic acid contents of *C. salviifolius* were higher than the other samples. Thus, the higher antioxidant potential of the *C. salviifolius* extracts may be related to its phenolic contents.

Moreover, the reduction in antioxidant potentials, inhibitory activities on carbohydrate-related enzymes and AGEs of the bioavailable samples may be related to the decline in marker flavonoid glycosides. However, *Cistus* extracts also contained other phenolic substances, as indicated in Figure 1. Therefore, a detailed chromatographical analysis of the extracts is required to monitor the influence of digestion on biological activity.

The inhibitory potential of plant extracts on digestive enzymes has recently attracted more attention due to the safety concern of synthetic inhibitors. Therefore, the inhibitory effect of plant extracts was determined in several studies, and this effect was generally associated with phenolic substances such flavonoids, phenolic acids, proanthocyanidins, etc. Sun et al. [37] suggested that polyphenolic compounds display their inhibitory effects by binding with the enzymes mentioned above with the help of hydrophobic forces and non-covalent bonds. Therefore, inhibition of α-amylase and α-glucosidase enzyme activity by polyphenols is related to their molecular structures. While this interaction mechanism has been studied using different techniques such as inhibition kinetics, molecular docking, fluorescence quenching, etc., no certain conclusion has been acquired yet [38]. However, numerous studies have reported that digestive enzyme inhibitions of the plant extracts are directly related to their phenolic contents. Similar studies on the enzyme inhibitory activities of *Cistus* species have also been previously reported. Sayah et al. [39] investigated the α-amylase and α-glucosidase inhibitory activities of the 80% methanolic and aqueous extracts from *C. monspeliensis* and *C. salviifolius*. Their results were in accordance with the present study, revealing that *C. salviifolius* aqueous extract demonstrated higher α-glucosidase (IC_50_ μg/mL: 0.95 ± 0.14) and α-amylase (IC_50_ μg/mL: 217.1 ± 0.15) inhibitory activity than the *C. monspeliensis* aqueous extract (IC_50_ μg/mL: 14.58 ± 1.26) and (IC_50_ μg/mL: 886.10 ± 0.10), respectively. The inhibitory rates of both aqueous extracts on these enzymes were higher than the reference compound acarbose (IC_50_ μg/mL: 18.01 ± 2.00). Similar to our study, they found correlation with the enzyme inhibitory rates and the total phenolic and flavonoid amounts. Both total phenolic and total flavonoid contents of the *C. salviifolius* aqueous extract (408.43 ± 1.09 mg GAE and 140.00 ± 1.15 RE, respectively) were higher than the *C. monspeliensis* aqueous extract (261.76 ± 1.9 mg GAE and 78.00 ± 1.15 RE, respectively). Orhan et al. [40] also investigated the digestive enzyme inhibitory potentials of 80% aqueous and ethanolic extracts from the leaves of *C. laurifolius*. According to their results, 80% ethanolic extract (71.7% ± 0.6) displayed a strong α-amylase inhibitory activity compared to aqueous extract (39.3% ± 2.2) at 1 mg/mL concentration. They suggested that phenolic compounds, especially flavonoids, directly affect insulin secretion by preventing beta-cell apoptosis and support anti-diabetic activity. This hypothesis, correlated with our results, marks that ND sample of aqueous extracts of *C. salviifolius* exerted the highest total flavonoid and phenolic contents and the greatest α-amylase and α-glucosidase inhibitory activities. As given in Table 4, aqueous extract of *C. salviifolius* exhibited better inhibitory activities on digestive enzymes than the other extracts.

Additionally, IN samples of the aqueous extracts contained lower phenolic and flavonoid amounts than ND samples and thus revealed lower enzyme inhibitory activities in the present work. While phenolic contents of the extracts were negatively affected by the digestion procedure, they still exhibited significant digestive enzyme inhibitory activity. In several studies, flavonoid glycosides were reported as the major metabolites of plant extracts with strong α-amylase and α-glucosidase inhibitory potentials [41,42,43]. While the inhibitory mechanism of phenolic compounds on the digestive enzymes has not been revealed yet, structure–activity relationship studies have been carried out on some phenolic components such as flavonoids, phenolic acids, proanthocyanidins and tannins. Structure–activity relationship studies about flavonoids showed that C2=C3 double bond of C-ring enhances the digestive enzyme inhibition potential of such compounds. Since this bond elevates the electron density, the strength of interaction between the flavonoid and enzyme is increased [44]. In addition, hydroxyl groups on C-5 and C-7 facilitate α-amylase inhibitory potential of flavonoids. It was also suggested that hydroxylation of flavonoids skeleton positively affects their α-amylase and α-glucosidase enzyme inhibitory potentials [45]. As indicated earlier, all marker flavonoids in the present study were flavonol glycosides bearing these structural requirements described above. However, after in vitro digestion procedure, their related activities declined significantly in bioavailable samples of the aqueous extracts. 

Generally, the inhibitory potential of the plant extracts on AGEs is related to several factors, i.e., their phenolic contents, antioxidant potentials, metal chelating capabilities, protein interactions and AGE receptor blocking activities [13]. As presented in Table 4, bioavailable samples of the aqueous extracts displayed lower AGE inhibitory activities compared to ND samples since in vitro digestion procedure adversely affected the phenolics content of the extracts. According to the current study outcomes, the ND sample of aqueous extract of *C. salviifolius* possessed the highest AGE inhibitory activity as well as total phenolic and flavonoid contents. In comparison, IN sample of aqueous extract of *C. monspeliensis* showed the weakest AGE inhibition potential, which may be ascribed to its lowest total flavonoid and phenolic contents. Since flavonoids have a widespread distribution in plant extracts, fruits, vegetables and beverages, several studies have intensified on inhibition potential of flavonoids on AGE formations. The same flavonoids assigned as the marker phenolics in this study were also reported as the marker compounds in other studies [46,47,48].

Similarly to digestive enzyme inhibition, AGE inhibitory activities of flavonoids were stimulated by C2=C3 double bond and hydroxylation of A and C rings. However, sugar attachment to the flavonoid skeleton leads to decreased inhibitory activity [49]. On the other hand, Cervantes-Lauren et al. [50] suggested that flavon-3-ol glycosides possessed a greater AGE inhibition potential than the other flavonoid glycosides. This hypothesis may be an explanation for the decreased AGE inhibition in bioavailable samples. For instance, quercitrin and hyperoside were not detected in bioavailable samples of the aqueous extract of *C. monspeliensis*, which is the reason for the lower activity of IN samples of *C. monspeliensis* compared to the extracts from other species. Even though quercitrin was not detected in the aqueous extract of *C. salviifolius*, significantly higher amounts of hyperoside and tiliroside may be the cause of its high AGE inhibition activity. In general, a positive correlation was observed between the marker flavonoid contents of the samples and their inhibitory potentials on AGEs. 

## 4. Material and Methods

### 4.1. Chemicals

All references, enzymes and chemicals employed in the experiments were purchased from Sigma Chemical Co. (St. Louis, MO, USA). Analytical grade materials were used in the experiments.

### 4.2. Plant Samples

Aerial parts of *C. creticus* and *C. salviifolius* were gathered from the campus of Yeditepe University (Kayışdağı, İstanbul) during the last week of April 2018. Aerial parts of *C. monspeliensis* and *C. parviflorus* were gathered from near Alaçatı Kutlu Aktaş Barajı, Çeşme, İzmir in the second week of May 2018. Aerial parts of *C. laurifolius* were collected from Kemer-Doğanhisar road, Konya, in the first week of June 2018. Prof. Dr. Erdem Yesilada authenticated plant materials. Voucher specimens for *C. creticus* (YEF18013), *C. laurifolius* (YEF18017), *C. monspeliensis* (YEF18015), *C. parviflorus* (YEF18016) and *C. salviifolius* (YEF18014) were stored at the Herbarium of the Department of Pharmacognosy, Faculty of Pharmacy, Yeditepe University, İstanbul, Turkey.

### 4.3. Extraction Procedure

Aqueous extraction was chosen since it is commonly used as a preparation technique in traditional medicine. The coarsely air dried and powdered aerial parts of *Cistus* species (100 g) were extracted with hot distilled water (80 °C, 1.5 L) by using a shaking device for 15 min. Then, aqueous extracts were filtered through a filter paper, evaporated to dryness under reduced pressure. After the lyophilisation procedure was completed, extracts were dissolved in distilled water for further processing (non-digested sample: ND) (the yield of extracts: 13.04% for *C. creticus*, 15.7% for *C. laurifolius*, 12.8% for *C. monspeliensis*, 14.94% for *C. parviflorus*, 14.74% for *C. salviifolius*).

### 4.4. In Vitro Human Digestion Simulation Method

The human digestion simulation model was applied to *Cistus* samples in vitro, following the method detailed earlier by Celep et al. [34]. Firstly, 1 g NaCl and 1.6 g pepsin were dissolved in 500 mL of distilled water to obtain a simulated gastric fluid solution (SGF). Later, the pH of SGF solution was arranged to 2 with HCl (5 M); 17.5 mL of this solution was mixed with 2.5 mL of plant samples, and this mixture was situated into the shaking water bath at 37 °C for 2 h to imitate the peristaltic movements of the digestion system. After 2 h, the samples were put into an ice bath to inactivate the enzymatic reactions; 2 mL of the samples was set aside as a “post-gastric” (PG) sample for further experiments. Cellulosic dialysis membrane loaded with a proper amount of NaHCO_3_ (1 M, pH 7) was located in the cold sample solutions; thus, gastrointestinal absorption was mimicked. Then, 4.5 mL of bile acids/pancreatin solution was combined with the solutions, and the mixture was incubated for another 2 h at 37 °C. Finally, the fluid inside the dialysis membrane was acquired as the “bioavailable” sample (IN). After the procedure was over, all samples were preserved at −20 °C for further experiments.

### 4.5. In Vitro Estimation of Phenolic Profile

#### 4.5.1. Total Phenolic Content Assay

Spectrophotometric determination of the total phenolic content of the samples was conducted in a 96-well plate template according to the method detailed earlier by Barak et al. [32]; 75 μL of Na_2_CO_3_ (20% in H_2_O) was added to 20 μL of freshly prepared sample and reference solutions. Then, 100 μL of Folin–Ciocalteu reagent was combined with the mixture. After a 30 min incubation period at room temperature in the dark, the absorbance was measured at 690 nm. Gallic acid was used as a reference solution at different concentrations to establish a calibration curve and total phenolic contents were expressed as gallic acid equivalents (GAE).

#### 4.5.2. Total Flavonoid Content Assay

Spectrophotometric determination of total flavonoid content of the samples was managed in a 96-well plate template in accordance with the previously explained method by Bardakci et al. [51]; 150 μL of 75% ethanol, 10 μL of aluminum chloride and 10 μL of 1M sodium acetate trihydrate were combined with 50 μL of sample and reference solutions, separately. Then, these mixtures were incubated at darkroom temperature for 30 min. After the incubation period, absorbance was calculated at 405 nm. Quercetin was employed as a reference solution at different concentrations to establish a calibration curve and total flavonoid contents were represented as quercetin equivalents (QE).

#### 4.5.3. Total Phenolic Acid Content Assay

The total phenolic acid content of the samples was measured spectrophotometrically following the procedure reported formerly by Barak et al. [52]. Firstly, proper amounts of sodium nitrite and sodium molybdate were dissolved in distilled water to obtain Arnow reagent. Then, 1 mL of the samples was combined with 1 mL of Arnow reagent, 1 mL of 0.1 M HCl and 1 mL of 1 M NaOH, separately. After that, the volume of the mixture was adjusted to 10 mL with distilled water, and the absorbance was read immediately at 490 nm. Caffeic acid was employed as a reference solution at different concentrations to get a calibration curve, and total phenolic acid contents were given as caffeic acid equivalents (CAE).

#### 4.5.4. Total Proanthocyanidin Content Assay

Spectrophotometric determination of the total proanthocyanidin content of the samples was conducted in a 96-well plate template by following the method of Barak et al. [11]. Briefly, 25 µL of the sample solutions was mixed with 150 µL of 4% vanillin and 75 µL of HCl solutions (32%), respectively. After 15 min incubation time at darkroom temperature, the absorbance was adjusted to 492 nm. Catechin hydrate was utilised as a reference solution at different concentrations to obtain a calibration curve. Methanol was employed as a control solution. The total proanthocyanidin content of the samples was stated as catechin equivalents (CE).

### 4.6. Free Radical Scavenging Activity Assays

#### 4.6.1. DPPH Radical Scavenging Activity Assay

DPPH radical scavenging activity of the samples was determined in a 96-well plate template following the method modified by Celep et al. [53]. At first, 150 µM of DPPH solution was freshly prepared. Then, 200 μL of DPPH solution was mixed with 25 µL of sample solutions. Then, this mixture was incubated at darkroom temperature for 50 min. The absorbance was calculated at 540 nm. Butylated hydroxytoluene (BHT) was employed as a reference solution at different concentrations. Methanol was used as a control solution. The activity of samples was presented as EC_50_, corresponding to the concentration showing 50% activity.

#### 4.6.2. DMPD Radical Scavenging Activity Assay

DMPD^+^ (N,N-dimethyl-p-phenylendiamine) radical scavenging activity of the samples was carried out in a 96-well plate template according to the method described earlier by İnan et al. [13]. Firstly, 100 mM DMPD^+^ solution, 0.05 M FeCl_3_·6H_2_O solution and 0.01 M acetate buffer were freshly prepared. Then, 1 mL of DMPD solution, 100 mL of acetate buffer and 0.2 mL of FeCl_3_·6H_2_O solution were mixed, and later 15 µL of sample solutions were combined with 210 µL of this mixture. After the incubation period at darkroom temperature for 50 min, the absorbance was measured at 492 nm. Trolox was used as a reference solution at different concentrations to obtain a calibration curve. Concentrations of the sample solutions were 1 mg/mL. DMPD radical scavenging activities of the samples were stated as Trolox equivalents (TE).

### 4.7. Metal Reducing Activity Assays

#### 4.7.1. Ferric Reducing Antioxidant Power Assay (FRAP)

Determination of FRAP activity of the samples was accomplished in a 96 well-plate template following the procedure reported earlier by Bardakci et al. [54]. At the beginning of the experiment, FRAP reagent was formed by mixing acetate buffer, ferric-tripyridyltriazine and FeCl_3_·6H_2_O solutions. After that, FRAP reagent was put inside the 37 °C oven for 30 min. Then, 10 µL of the sample solutions was combined with 30 μL of distilled water and 260 μL of FRAP reagent, respectively. After incubation time at 37 °C for 30 min., the absorbance was adjusted to 593 nm. A standard curve was built by employing different molarities of ferrous sulfate (0.25–2 mM) solution to evaluate the results. BHT was used as a reference solution at different concentrations. FRAP activities of the samples were presented as mM FeSO_4_ in 1 g dry extract.

#### 4.7.2. Cupric Reducing Antioxidant Capacity Assay (CUPRAC)

CUPRAC activity of the samples was determined in a 96-well plate template following the method modified by Celep et al. [31]; 85 μL of CuSO_4_ (10 mM), neocupraine and ammonium acetate solutions and 51 uL of distilled water were added to 43 uL of sample solutions, separately. Following an incubation period (20 min) at 50 °C in a water bath, the absorbance was measured at 450 nm. Ascorbic acid was used as a reference solution at different concentrations to acquire a calibration curve. CUPRAC activities of the samples were presented as ascorbic acid equivalents (AAE).

### 4.8. Total Antioxidant Activity Assay (TOAC)

Total antioxidant activity determination of the samples was performed in a 96-well plate template following the procedure by Celep et al. [55]. At first, a certain amount of sodium phosphate monobasic, ammonium molybdate tetrahydrate and sulfuric acid were mixed to acquire TOAC solution. Then, 300 µL of TOAC solution was added to 30 μL of sample solutions. After the incubation period at 95 °C in a water bath for 90 min, the absorbance was determined at 690 nm. Ascorbic acid was used as a reference solution at different concentrations to acquire a calibration curve. TOAC activities of the samples were represented as ascorbic acid equivalents (AAE).

### 4.9. Estimation of Bioavailability Index

The bioavailability index (BAvI) was calculated according to the theoretical equation described by İnan et al. [13]:

BAvI = C*_IN_*/C*_ND_*

The “bioavailability index” (BAvI) was described as the ratio of the amount of phenolics in the bioavailable sample (*IN*) to that in the non-digested sample (*ND*).

### 4.10. Quantification of Marker Flavonoids by HPTLC

The quantitative determination of tiliroside, hyperoside and quercitrin concentrations in all simulation samples (ND, PG, IN) of the aqueous extracts from *Cistus* species was carried out by high-performance thin-layer chromatography (HPTLC) (CAMAG, Muttenz, Switzerland) following the method validated by Guzelmeric et al. [16]. Hyperoside, tiliroside and quercitrin were prepared in 25, 50 and 100 μg/mL concentrations and the concentrations of freshly prepared sample solutions were adjusted to 10 mg/mL. These solutions were applied to normal phase glass-backed silica gel plates (20 cm × 10 cm, Merck, Darmstadt, Germany) with certain volumes (1–5 μL standard solutions and 5 μL sample solutions) by using 100 μL syringes (Hamilton, Bonaduz, Switzerland). The application procedure was performed with Linomat 5 sample applicator. The development process was performed in Automated Development Chamber (ADC 2) and ethyl acetate:dichloromethane:acetic acid:formic acid:water (100:25:10:10:11 by volume) was selected as a mobile phase. Then, the plates were derivatised with Natural Product Reagent (NPR) (1g diphenylboric acid 2-aminoethylester in 200 mL of ethyl acetate) in the immersion device (CAMAG). While hyperoside and quercitrin were analysed spectrophotometrically at 260 nm, the amount of tiliroside was measured at 330 nm by UV scanner. R*f* values of the standards were determined as hyperoside (≈0.35), quercitrin (≈0.45), tiliroside (≈0.65). The correlation coefficients (r^2^) were found to be >0.98 for the quantification of the marker flavonoids.

### 4.11. Inhibitory Activity on Diabetes-Related Enzymes

#### 4.11.1. α-Glucosidase Inhibitory Activity

α-glucosidase inhibitory activities of the non-digested and bioavailable samples obtained from the aqueous extracts of *Cistus* species were examined following the method explained earlier by Balan et al. [56]. Firstly, proper amounts of monosodium phosphate and disodium phosphate were mixed with procuring 100 mM phosphate buffer (pH 7). α-glucosidase enzyme was dissolved in phosphate buffer to obtain the α-glucosidase solution (0.2 U/mL). Then 170 μL of phosphate buffer, 20 μL of α-glucosidase solution and 20 μL of sample solutions were combined and incubated in a 37 °C oven for 15 min. After that, 20 μL of 2.5 mM p-nitrophenyl-α-d-glucopyranoside solution in 100 mM potassium phosphate buffer (pH 7.0) was added to the mixture, and another incubation period was executed at 37 °C for 15 min. Then, 80 μL of 0.2 M sodium carbonate solution was appended to the mixture to terminate the reaction. Absorbance was measured at 405 nm. Quercetin solution at different concentrations was used as a reference. Results were estimated as the percentage of inhibitory activity in 1 mg/mL and 0.5 mg/mL concentrations of ND and IN samples of the aqueous extracts of *Cistus* species.

#### 4.11.2. α-Amylase Inhibitory Activity

α-amylase inhibitory activity of non-digested and bioavailable samples obtained from the aqueous extracts of *Cistus* species was determined by following the procedure detailed previously by Balan et al. [56]. Spectrophotometric determination of *α*-amylase inhibitory activities of the samples was performed by employing DNS (3,5-dinitrosalicylic acid) reagent. As stated in the method, maltose is formed from the conversion of the starch and the yellow color of alkaline DNS is turned into the orange-red color due to maltose produced from starch. Thus, 96 mM DNS solution was prepared from the mixture of sodium potassium tartrate solution (dissolved in 2 M NaOH) and a certain amount of DNS (dissolved in distilled water). Then, 20 mM sodium phosphate buffer with 6.7 mM NaCl (co-factor of *α*-amylase enzyme) was prepared at 20 °C (pH: 6.9). *α*-Amylase enzyme (1U/mL) and starch (10 mg/mL) were dissolved in this buffer. After that, 50 μL of sodium phosphate buffer and 10 μL of *α*-amylase enzyme solution were mixed with 20 μL of the sample solutions. This mixture was incubated at 37 °C for 45 min. After the incubation period, 20 μL of the starch solution was added to the mixture. Another incubation period was started at 37 °C for 45 min. The same procedure was applied to the samples without the addition of *α*-amylase enzyme solution called “sample background”. The control group was studied with the same procedure in the absence of sample solutions. Absorbance was measured at 540 nm. Acarbose was used as a reference solution at different concentrations. Results were presented as a percentage of inhibitory activity in 1 mg/mL and 0.5 mg/mL concentrations of ND and IN samples from aqueous extracts of *Cistus* species.

#### 4.11.3. AGE Inhibitory Activity

AGE inhibitory activity of non-digested and bioavailable samples from aqueous extracts of *Cistus* species was determined following the method described by Starowicz et al. [57]. Before any process, 1 mg/mL and 0.5 mg/mL concentrations of ND and IN samples were freshly prepared. Then, 1 mL of 10 mg/mL concentration of bovine serum albumin (BSA) solution was added to 1 mL of ND and IN sample solutions. Control samples were prepared without adding ND and IN sample solutions, and the blank samples were prepared without adding 0.5 M glucose. Then, all prepared samples were incubated for 40 h in a shaking water bath at 55 °C. After the incubation period ended, Thermo Scientific™ Varioskan™ LUX multimode microplate reader was used in the 370 nm excitation/440 nm emission range to calculate fluorescence intensity. Quercetin was used as a reference solution at different concentrations.

### 4.12. Statistical Analysis

All experiments were performed independently three different times. The GraphPad Prism software program (6.1 version) was utilised to determine the parametric or non-parametric distribution of the data. One way ANOVA Tukey’s Multiple comparisons analysis section was used to evaluate TPC, TFC, TPAC, TSC, TPACC, FRAP, CUPRAC, TOAC, DPPH, DMPD assays. On the other hand, the results of α-amylase, α-glucosidase and AGE inhibition experiments were examined by two-way ANOVA Sidak’s multiple comparisons analysis. Significant results were shown with *p* < 0.05.

## 5. Conclusions

In the present study, the aqueous extracts of all *Cistus* species recorded in Turkish flora were investigated for their phenolic profiles and in vitro antioxidant and antidiabetic potentials. Since decoction or infusion is the common form in traditional medicines, using aqueous extracts for the activity assessment in experimental studies is particularly important. On the other hand, hydrophilic constituents in the aqueous extract are subjected to a serial of metabolic transformations in the gastrointestinal system once ingested. Therefore for correct activity evaluation of traditional formulations, activities of the bioavailable metabolites should also be investigated.

This is the first study to examine the consequences of the in vitro human digestion simulation method on Turkish *Cistus* species. In addition, the inhibition potential of Turkish *Cistus* species on AGEs was studied in this study for the first time. Furthermore, tiliroside, hyperoside and quercitrin were assigned marker flavonoids, and alterations in their concentrations were monitored in the in vitro digestion procedure. While phenolic contents and antidiabetic and antioxidant activities of the extracts were negatively affected by gastrointestinal digestion procedures, they still exhibited significant bioactivity. According to the results, *C. salviifolius* extract was detected as the most potent plant in terms of phenolic content and antioxidant and anti-diabetic activities. In conclusion, aerial parts from Turkish *Cistus* species have rich phenolic contents and potential antioxidant and anti-diabetic activities. While the in vitro digestion simulation method was employed to evaluate bioavailability, it might not fully mimic the metabolic pathways occurring in the organism. Therefore, further in vivo and clinical studies are required to assess the bioavailability of the phenolic compounds and their contribution to the reported pharmacological effects in detail.

## Figures and Tables

**Table 1:** Spectrophotometric determination of phenolic profile of *Cistus* extracts and their bioavailability indexes.

Assays	Samples *	ND ^A^	PG	IN	BAvI (%)
Total phenolic content ^B^	CCA	275.47 ^a^ ± 6.86	149.59 ^b^ ± 4.95	23.77 ^c^ ± 0.68	8.6%
	CLA	289.15 ^a^ ± 4.99	145.23 ^b^ ± 3.99	21.59 ^c^ ± 0.74	6.4%
	CMA	257.91 ^a^ ± 5.01	142.24 ^b^ ± 5.19	21.36 ^c^ ± 0.94	8.3%
	CPA	330.18 ^a^ ± 1.87	161.51 ^b^ ± 4.86	26.46 ^c^ ± 0.74	8.01%
	CSA	430.47 ^a^ ± 6.45	257.53 ^b^ ± 0.63	29.59 ^c^ ± 0.74	6.87%
Total flavonoid content ^C^	CCA	24.15 ^a^ ± 1.98	22.86 ^a^ ± 1.12	4.92 ^b^ ± 0.48	20.37%
	CLA	22.98 ^a^ ± 1.38	21.87 ^a^ ± 1.17	4.1 ^b^ ± 0.77	17.84%
	CMA	19.21 ^a^ ± 1.23	17.78 ^a^ ± 0.96	2.97 ^b^ ± 0.57	15.46%
	CPA	32.17 ^a^ ± 1.96	33.75 ^a^ ± 1.45	5.9 ^b^ ± 0.43	18.34%
	CSA	60.25 ^a^ ± 2.19	54.73 ^a^ ± 1.89	14.2 ^b^ ± 0.92	23.5%
Total phenolic acid content ^D^	CCA	46.18 ^a^ ± 0.61	47.07 ^a^ ± 0.25	16.03 ^b^ ± 0.54	34.71%
	CLA	44.39 ^a^ ± 0.58	42.46 ^a^ ± 1.01	15.29 ^b^ ± 1.26	34.45%
	CMA	44.75 ^a^ ± 1.01	42.96 ^a^ ± 1.02	13.25 ^b^ ± 0.72	38.55%
	CPA	50.64 ^a^ ± 0.76	47.54 ^a^ ± 1.52	19.01 ^b^ ± 1.26	37.53%
	CSA	52.93 ^a^ ± 1.07	50.29 ^a^ ± 0.76	20.28 ^b^ ± 0.81	38.29%
Total proanthocyanidin content ^E^	CCA	61.08 ^a^ ± 2.81	17.79 ^b^ ± 0.72	N.D.	-
	CLA	59.67 ^a^ ± 4.38	18.71 ^b^ ± 5.91	N.D.	-
	CMA	29.33 ^a^ ± 3.08	9.12 ^b^ ± 4.38	N.D.	-
	CPA	38.91 ^a^ ± 3.13	13.21 ^b^ ± 1.81	N.D.	-
	CSA	45.74 ^a^ ± 0.72	16.83 ^b^ ± 1.57	N.D.	-

^A^ The abbreviations for samples are ND: Non-digested; PG: Postgastric; IN: Bioavailable; BAvI: Bioavailability index; ^B^ Results were stated as the mean of triplicates ± standard deviation (S.D.) and as mg gallic acid equivalents (GAE) in 1 g sample; ^C^ Results were expressed as the mean of triplicates ± standard deviation (S.D.) and as mg quercetin equivalents (QE) in 1 g sample; ^D^ Results were expressed as the mean of triplicates ± standard deviation (S.D.) and as mg caffeic acid equivalents (CAE) in 1 g sample; ^E^ Results were expressed as the mean of triplicates ± standard deviation (S.D.) and as mg catechin equivalent (CE) in 1 g sample; * Abbreviations of the aqueous extracts: CCA for *C. creticus*, CLA for *C. laurifolius*, CMA for *C. monspeliensis,* CPA for *C. parviflorus*, CSA for *C. salviifolius*. Different letters in the same row indicate significance (*p* < 0.05).

**Table 2:** Quantification of the marker flavonoids by HPTLC and their bioavailability indexes.

Assays	Samples *	ND ^A^	PG	IN	BAvI (%)
Tiliroside ^B^	CCA	2.2 ^a^ ± 0.22	1.9 ^b^ ± 0.11	0.35 ^c^ ± 0.04	15.9%
	CLA	1.71 ^a^ ± 0.17	1.35 ^b^ ± 0.15	0.43 ^c^ ± 0.08	25.15%
	CMA	1.05 ^a^ ± 0.19	0.93 ^a^ ± 0.11	ND	-
	CPA	2.84 ^a^ ± 0.21	2.82 ^a^ ± 0.23	0.66 ^b^ ± 0.06	23.24%
	CSA	3.76 ^a^ ± 0.24	3.17 ^b^ ± 0.21	1.01 ^c^ ± 0.11	26.86%
Hyperoside	CCA	1.64 ^a^ ± 0.14	1.45 ^a^ ± 0.13	0.41 ^b^ ± 0.04	25%
	CLA	2.05 ^a^ ± 0.24	1.96 ^a^ ± 0.19	0.51 ^b^ ± 0.07	24.88%
	CMA	0.71 ^a^ ± 0.1	0.66 ^a^ ± 0.08	ND	-
	CPA	1.67 ^a^ ± 0.15	1.54 ^a^ ± 0.12	0.43 ^b^ ± 0.03	25.75%
	CSA	4.59 ^a^ ± 0.31	4.25 ^a^ ± 0.26	1.16 ^b^ ± 0.16	25.27%
Quercitrin	CCA	3.05 ^a^ ± 0.26	2.72 ^b^ ± 0.21	0.74 ^c^ ± 0.11	24.26%
	CLA	0.97 ^a^ ± 0.12	0.76 ^b^ ± 0.11	ND	-
	CMA	0.98 ^a^ ± 0.14	0.86 ^a^ ± 0.12	0.21 ^b^ ± 0.03	21.43%
	CPA	2.73 ^a^ ± 0.24	2.38 ^a^ ± 0.23	0.71 ^b^ ± 0.08	26%
	CSA	ND	ND	ND	-

^A^ The abbreviations for samples are ND: Non-digested; PG: Postgastric; IN: Bioavailable; BAvI: Bioavailability index; ^B^ Results were given as mg/g dry extract and experiments were performed independently three different times; * Abbreviations of the aqueous extracts: CCA for *C. creticus*, CLA for *C. laurifolius*, CMA for *C. monspeliensis*, CPA for *C. parviflorus*, CSA for *C. salviifolius*. Different letters in the same row indicate significance (*p* < 0.05).

**Table 3:** Spectrophotometric determination of antioxidant activities of *Cistus* extracts.

Assays	Samples *	ND ^A^	PG	IN
DPPH scavenging act. ^B^	CCA	5.63 ^a^ ± 0.53	5.55 ^a^ ± 0.53	10.83 ^b^ ± 0.46
	CLA	5.55 ^a^ ± 1.42	5.52 ^a^ ± 0.53	11.05 ^b^ ± 0.27
	CMA	5.57 ^a^ ± 0.94	5.53 ^a^ ± 0.44	13.75 ^b^ ± 0.31
	CPA	5.55 ^a^ ± 0.41	5.51 ^a^ ± 0.52	9.93 ^b^ ± 0.83
	CSA	5.55 ^a^ ± 0.58	5.74 ^a^ ± 0.02	8.73 ^b^ ± 0.58
DMPD scavenging act. ^C^	CCA	11.82 ^a^ ± 0.86	10.11 ^a^ ± 0.46	22.06 ^b^ ± 0.74
	CLA	10.43 ^a^ ± 0.97	9.42 ^a^ ± 0.67	26.08 ^b^ ± 0.12
	CMA	10.98 ^a^ ± 0.74	9.77 ^a^ ± 0.99	22.88 ^b^ ± 0.41
	CPA	10.22 ^a^ ± 0.25	9.41 ^a^ ± 0.23	18.48 ^b^ ± 0.45
	CSA	10.69 ^a^ ± 0.89	11.05 ^a^ ± 0.68	25.14 ^b^ ± 0.99
FRAP activity ^D^	CCA	5.99 ^a^ ± 0.13	2.92 ^b^ ± 0.06	0.90 ^c^ ± 0.01
	CLA	4.27 ^a^ ± 0.17	2.77 ^b^ ± 0.05	0.68 ^c^ ± 0.01
	CMA	4.60 ^a^ ± 0.07	2.97 ^b^ ± 0.15	0.52 ^c^ ± 0.03
	CPA	5.23 ^a^ ± 0.28	3.15 ^b^ ± 0.20	0.71 ^c^ ± 0.03
	CSA	7.52 ^a^ ± 0.69	4.44 ^b^ ± 0.16	0.69 ^c^ ± 0.02
CUPRAC activity ^E^	CCA	687.34 ^a^ ± 8.86	616.33 ^a^ ± 5.05	188.61 ^b^ ± 5.35
	CLA	627.39 ^a^ ± 5.89	608.98 ^a^ ± 6.53	121.03 ^b^ ± 2.24
	CMA	667.41 ^a^ ± 9.33	643.91 ^a^ ± 6.11	146.94 ^b^ ± 2.81
	CPA	688.76 ^a^ ± 4.31	625.16 ^a^ ± 5.30	147.62 ^b^ ± 5.97
	CSA	691.48 ^a^ ± 5.18	674.43 ^a^ ± 2.53	153.15 ^b^ ± 2.61
Total antioxidant capacity ^F^	CCA	208.38 ^a^ ± 3.48	117.96 ^b^ ± 5.31	41.88 ^c^ ± 1.77
	CLA	272.13 ^a^ ± 5.63	131.50 ^b^ ± 5.01	31.25 ^c^ ± 1.65
	CMA	236.18 ^a^ ± 6.63	195.88 ^b^ ± 2.65	30.18 ^c^ ± 1.33
	CPA	225.25 ^a^ ± 5.34	153.80 ^b^ ± 4.61	45.63 ^c^ ± 2.72
	CSA	222.55 ^a^ ± 3.77	167.54 ^b^ ± 5.01	49.29 ^c^ ± 2.37

^A^ The abbreviations for samples are ND: Non-digested; PG: Postgastric; IN: Bioavailable; BAvI: Bioavailability index; ^B^ Results were presented as EC_50_ in μg/mL equivalents and experiments were performed independently three different times. EC_50_ value of the reference compound “BHT” in DPPH scavenging activity was determined as 5.83 ± 0.2 μg/mL; ^C^ Results were presented as EC_50_ in μg/mL equivalents and experiments were performed independently three different times. EC_50_ value of the reference compound Trolox in DMPD scavenging activity was determined as 5.82 ± 0.37 μg/mL; ^D^ Results were expressed as mM FeSO4 equivalents in 1 g sample and experiments were performed independently three different times. FRAP activity of the reference compound “BHT” was detected as 4.06 ± 0.42 mM FeSO4 eq. in 1 g sample; ^E^ Results were given as mg ascorbic acid equivalent (AAE) in 1 g sample and experiments were performed independently three different times; ^F^ Results were given as mg ascorbic acid equivalent (AAE) in 1 g sample and experiments were performed independently three different times; * Abbreviations of the aqueous extracts: CCA for *C. creticus*, CLA for *C. laurifolius*, CMA for *C. monspeliensis*, CPA for *C. parviflorus*, CSA for *C. salviifolius*. Different letters in the same row indicate significance (*p* < 0.05).

**Table 4:** Spectrophotometric determination of antidiabetic activities of *Cistus* extracts.

Assays	Samples *	*ND* 0.5 mg/mL ^A^	ND 1mg/mL	IN 0.5 mg/mL	IN 1mg/mL
α-amylase inhibitory act. ^B^	CCA	65.05 ^a^ ± 1.18	73.94 ^b^ ± 0.22	49.73 ^c^ ± 0.29	54.90 ^d^ ± 0.87
	CLA	63.25 ^a^ ± 0.18	75.20 ^b^ ± 0.11	45.49 ^c^ ± 0.42	51.02 ^d^ ± 0.14
	CMA	66.58 ^a^ ± 0.64	71.31 ^b^ ± 3.31	42.69 ^c^ ± 0.38	48.08 ^d^ ± 0.41
	CPA	67.66 ^a^ ± 0.70	75.89 ^b^ ± 0.62	50.78 ^c^ ± 2.54	56.24 ^d^ ± 0.85
	CSA	72.21 ^a^ ± 0.47	80.34 ^b^ ± 0.19	54.71 ^c^ ± 0.30	61.83 ^d^ ± 0.20
α-glucosidase inhibitory act. ^C^	CCA	64.11 ^a^ ± 1.03	71.62 ^b^ ± 1.48	47.83 ^c^ ± 0.23	56.33 ^d^ ± 0.55
	CLA	66.29 ^a^ ± 0.80	69.79 ^a^ ± 0.60	52.75 ^b^ ± 1.16	61.75 ^c^ ± 0.23
	CMA	58.48 ^a^ ± 0.85	62.37 ^b^ ± 1.74	41.72 ^c^ ± 1.88	45.14 ^d^ ± 0.57
	CPA	71.26 ^a^ ± 0.46	79.13 ^b^ ± 0.82	52.13 ^c^ ± 1.87	61.27 ^d^ ± 1.44
	CSA	76.25 ^a^ ± 1.95	87.12 ^b^ ± 0.73	63.66 ^c^ ± 1.63	67.60 ^d^ ± 0.96
AGEs inhibitory act. ^D^	CCA	85.38 ^a^ ± 1.57	93.03 ^b^ ± 2.26	68.01 ^c^ ± 5.63	73.11 ^d^ ± 2.38
	CLA	83.24 ^a^ ± 1.03	87.96 ^b^ ± 2.58	64.96 ^c^ ± 1.96	72.07 ^d^ ± 4.78
	CMA	77.02 ^a^ ± 3.56	82.62 ^b^ ± 3.05	56.60 ^c^ ± 4.15	69.60 ^d^ ± 3.36
	CPA	85.04 ^a^ ± 2.95	92.65 ^b^ ± 1.41	71.54 ^c^ ± 2.27	81.10 ^d^ ± 3.76
	CSA	90.19 ^a^ ± 2.62	98.39 ^b^ ± 1.25	76.71 ^c^ ± 4.15	84.26 ^d^ ± 3.33

^A^ The abbreviations for samples are ND: Non-digested; PG: Postgastric; IN: Bioavailable; BAvI: Bioavailability index; ^B^ Results were stated as the mean of triplicates ± standard deviation (S.D.) and acarbose was used as control group with 75.8 ± 0.02% inhibition at 1 mg/mL, 65.45 ± 0.01% inhibition at 0.5 mg/mL; ^C^ Results were expressed as the mean of triplicates ± standard deviation (S.D.) and quercetin was used as control group with 80.4 ± 0.03% inhibition at 1 mg/mL, 69.66 ± 0.05% inhibition at 0.5 mg/mL; ^D^ Results were expressed as the mean of triplicates ± standard deviation (S.D.) and quercetin was used as control group with 89.33 ± 3.47% inhibition at 1 mg/mL, 72.03 ± 3.04% inhibition at 0.5 mg/mL; * Abbreviations of the aqueous extracts: CCA for *C. creticus*, CLA for *C. laurifolius*, CMA for *C. monspeliensis*, CPA for *C. parviflorus*, CSA for *C. salviifolius*. Different letters in the same row indicate significance (*p* < 0.05).

## Data Availability

The data presented in this study are available on request from the corresponding author.

## Sample Availability

Samples of the compounds are not available from the authors.

## References

[1] Küpeli E., Yesilada E. (2007). Flavonoids with anti-inflammatory and antinociceptive activity from *Cistus laurifolius* L. leaves through bioassay-guided procedures. J. Ethnopharmacol..

[2] Kalus U., Grigorov A., Kadecki O., Jansen J.P., Kiesewetter H., Radtke H. (2009). *Cistus incanus* (CYSTUS052) for treating patients with infection of the upper respiratory tract. A prospective, randomised, placebo-controlled clinical study. Antivir. Res..

[3] Akkol E.K., Orhan I.E., Yesilada E. (2012). Anticholinesterase and antioxidant effects of the ethanol extract, ethanol fractions and isolated flavonoids from *Cistus laurifolius* L. leaves. Food Chem..

[4] Barrajón-Catalán E., Fernández-Arroyo S., Saura D., Guillén E., Fernández-Gutiérrez A., Segura-Carretero A., Micol V. (2010). Cistaceae aqueous extracts containing ellagitannins show antioxidant and antimicrobial capacity, and cytotoxic activity against human cancer cells. Food Chem. Toxicol..

[5] Tomás-Menor L., Morales-Soto A., Barrajón-Catalán E., Roldán-Segura C., Segura-Carretero A., Micol V. (2013). Correlation between the antibacterial activity and the composition of extracts derived from various Spanish *Cistus* species. Food Chem. Toxicol..

[6] Coode M., Davis PH (1965). Flora of Turkey and the Aegean Islands.

[7] Polat R., Satil F. (2012). An ethnobotanical survey of medicinal plants in Edremit Gulf (Balikesir - Turkey). J. Ethnopharmacol..

[8] Gürdal B., Kültür Ş. (2013). An ethnobotanical study of medicinal plants in Marmaris (Muǧla, Turkey). J. Ethnopharmacol..

[9] Honda G., Yeşilada E., Tabata M., Sezik E., Fujita T., Takeda Y., Takaishi Y., Tanaka T. (1996). Traditional medicine in Turkey VI. Folk medicine in West Anatolia: Afyon, Kütahya, Denizli, Muğla, Aydin provinces. J. Ethnopharmacol..

[10] Alfadda A.A., Sallam R.M. (2012). Reactive oxygen species in health and disease. J. Biomed. Biotechnol..

[11] Barak T.H., Celep E., İnan Y., Yesilada E. (2019). Influence of in vitro human digestion on the bioavailability of phenolic content and antioxidant activity of *Viburnum opulus* L. (European cranberry) fruit extracts. Ind. Crops Prod..

[12] Lee O.N., Ak G., Zengin G., Cziáky Z., Jekő J., Rengasamy K.R.R., Park H.Y., Kim D.H., Sivanesan I. (2020). Phytochemical composition, antioxidant capacity, and enzyme inhibitory activity in callus, somaclonal variant, and normal green shoot tissues of *Catharanthus roseus* (L) G. Don. Molecules.

[13] İnan Y., Kurt-Celep I., Akyüz S., Barak T.H., Celep E., Yesilada E. (2020). An investigation on the enzyme inhibitory activities, phenolic pro fi le and antioxidant potentials of *Salvia virgata* Jacq. S. Afr. J. Bot..

[14] Zengin G., Sieniawska E., Senkardes I., Picot-Allain M.C.N., Ibrahime Sinan K., Fawzi Mahomoodally M. (2019). Antioxidant abilities, key enzyme inhibitory potential and phytochemical profile of *Tanacetum poteriifolium* Grierson. Ind. Crops Prod..

[15] Sęczyk Ł., Sugier D., Świeca M., Gawlik-Dziki U. (2021). The effect of in vitro digestion, food matrix, and hydrothermal treatment on the potential bioaccessibility of selected phenolic compounds. Food Chem..

[16] Guzelmeric E., İnan Y., Yüksel P.I., Yesilada E. (2020). Laden bitkisinin (*Cistus creticus* L.) topraküstü kısımlarının standardizasyonunda kullanılan farmakognozik yöntemler. Türk Farmakop. Derg..

[17] Stumvoll M., Goldstein B.J., van Haeften T.W. (2005). Pathogenesis of type 2 diabetes. Lancet.

[18] Warren F.J., Fukuma N.M., Mikkelsen D., Flanagan B.M., Williams B.A., Lisle A.T., Ó Cuív P., Morrison M., Gidley M.J. (2018). Food starch structure impacts gut microbiome composition. mSphere.

[19] Khan M., Liu H., Wang J., Sun B. (2020). Inhibitory effect of phenolic compounds and plant extracts on the formation of advance glycation end products: A comprehensive review. Food Res. Int..

[20] Xiong Y., Ng K., Zhang P., Warner R.D., Shen S., Tang H., Liang Z., Fang Z. (2020). In vitro α-glucosidase and α-amylase inhibitory activities of free and bound phenolic extracts from the bran and kernel fractions of five sorghum grain genotypes. Foods.

[21] Wenzel U. (2013). Flavonoids as drugs at the small intestinal level. Curr. Opin. Pharmacol..

[22] da Costa Pinaffi A.C., Sampaio G.R., Soares M.J., Shahidi F., de Camargo A.C., Torres E.A.F.S. (2020). Insoluble-bound polyphenols released from guarana powder: Inhibition of alpha-glucosidase and proanthocyanidin profile. Molecules.

[23] Chen H., Virk M.S., Chen F. (2016). Phenolic acids inhibit the formation of advanced glycation end products in food simulation systems depending on their reducing powers and structures. Int. J. Food Sci. Nutr..

[24] Martineau L.C., Cuerrier A., Johns T., Haddad P.S., Bennett S.A.L. (2011). Inhibition of advanced glycation end product formation by medicinal plant extracts correlates with phenolic metabolites and antioxidant activity. Diabetes Metab. Syndr. Clin. Res. Rev..

[25] Asgharpour F., Ranjkesh Z., Taghi M. (2019). A systematic review of antiglycation medicinal plants. Diabetes Metab. Syndr. Clin. Res. Rev..

[26] Funke I., Melzig M. (2005). Effect of different phenolic compounds on α-amylase activity: Screening by microplate-reader based kinetic assay. Pharmazie.

[27] Lu Y., Franziska M., Song L. (2016). Oligomeric proanthocyanidins are the active compounds in *Abelmoschus esculentus* Moench for its α-amylase and α -glucosidase inhibition. J. Funct. Foods.

[28] Wu C.H., Yen G.C. (2005). Inhibitory effect of naturally occurring flavonoids on the formation of advanced glycation end products. J. Agric. Food Chem..

[29] Li J.K., Liu X.D., Shen L., Zeng W.M., Qiu G.Z. (2016). Natural plant polyphenols for alleviating oxidative damage in man: Current status and future perspectives. Trop. J. Pharm. Res..

[30] Hussain T., Tan B., Yin Y., Blachier F., Tossou M.C.B., Rahu N. (2016). Oxidative stress and inflammation: What polyphenols can do for us?. Oxid. Med. Cell. Longev..

[31] Celep E., Aydin A., Kirmizibekmez H., Yesilada E. (2013). Appraisal of in vitro and in vivo antioxidant activity potential of cornelian cherry leaves. Food Chem. Toxicol..

[32] Barak T.H., Celep E., İnan Y., Yeşilada E. (2020). In vitro human digestion simulation of the bioavailability and antioxidant activity of phenolics from *Sambucus ebulus* L. fruit extracts. Food Biosci..

[33] Karaś M., Jakubczyk A., Szymanowska U., Złotek U., Zielińska E. (2017). Digestion and bioavailability of bioactive phytochemicals. Int. J. Food Sci. Technol..

[34] Celep E., İnan Y., Akyüz S., Yesilada E. (2017). The bioaccessible phenolic profile and antioxidant potential of *Hypericum perfoliatum* L. after simulated human digestion. Ind. Crops Prod..

[35] Serra A., MacI A., Romero M.P., Anglés N., Morelló J.R., Motilva M.J. (2011). Metabolic pathways of the colonic metabolism of procyanidins (monomers and dimers) and alkaloids. Food Chem..

[36] Ketnawa S., Reginio F.C., Sukanya T., Ogawa Y. (2021). Changes in bioactive compounds and antioxidant activity of plant-based foods by gastrointestinal digestion: A review. Crit. Rev. Food Sci. Nutr..

[37] Sun L., Wang Y., Miao M. (2020). Inhibition of α-amylase by polyphenolic compounds: Substrate digestion, binding interactions and nutritional intervention. Trends Food Sci. Technol..

[38] Zhu J., Chen C., Zhang B., Huang Q. (2020). The inhibitory effects of flavonoids on α-amylase and α-glucosidase. Crit. Rev. Food Sci. Nutr..

[39] Sayah K., Marmouzi I., Naceiri Mrabti H., Cherrah Y., Faouzi M.E.A. (2017). Antioxidant activity and inhibitory potential of *Cistus salviifolius* (L.) and *Cistus monspeliensis* (L.) aerial parts extracts against key enzymes linked to hyperglycemia. Biomed. Res. Int..

[40] Orhan N., Aslan M., Şüküroǧlu M., Deliorman Orhan D. (2013). In vivo and in vitro antidiabetic effect of *Cistus laurifolius* L. and detection of major phenolic compounds by UPLC-TOF-MS analysis. J. Ethnopharmacol..

[41] Kim J. (2000). Inhibition of alpha-glucosidase and amylase by Luteolin, a Flavonoid. Biosci. Biotechnol. Biochem..

[42] Yuan E., Liu B., Wei Q., Yang J., Chen L., Li Q. (2014). Structure activity relationships of flavonoids as potent α-amylase Inhibitors. Nat. Prod. Commun..

[43] Grzegorczyk-Karolak I., Golab K., Gburek J., Wysokinska H., Matkowski A. (2016). Inhibition of advanced glycation end-product formation and antioxidant activity by extracts and polyphenols from *Scutellaria alpina* L. and *S. altissima* L.. Molecules.

[44] Martinez-Gonzalez A.I., Díaz-Sánchez G., de la Rosa L.A., Bustos-Jaimes I., Alvarez-Parrilla E. (2019). Inhibition of α-amylase by flavonoids: Structure activity relationship (SAR). Spectrochim. Acta-Part A Mol. Biomol. Spectrosc..

[45] Şöhretoğlu D., Sari S. (2020). Flavonoids as alpha-glucosidase inhibitors: Mechanistic approaches merged with enzyme kinetics and molecular modelling. Phytochem. Rev..

[46] Lee I.S., Kim Y.J., Jung S.H., Kim J.H., Kim J.S. (2017). Flavonoids from *Litsea japonica* inhibit AGEs formation and rat lense aldose reductase in vitro and vessel dilation in zebrafish. Planta Med..

[47] Zhang Z., Sethiel M.S., Shen W., Liao S., Zou Y. (2013). Hyperoside downregulates the receptor for advanced glycation end products (RAGE) and promotes proliferation in ECV304 cells via the c-Jun N-terminal Kinases (JNK) pathway following stimulation by advanced glycation end-products *in vitro*. Int. J. Mol. Sci..

[48] Yoon S.R., Shim S.M. (2015). Inhibitory effect of polyphenols in *Houttuynia cordata* on advanced glycation end-products (AGEs) by trapping methylglyoxal. LWT—Food Sci. Technol..

[49] Liu L., Zhang L., Ren L., Xie Y. (2020). Advances in structures required of polyphenols for xanthine oxidase inhibition. Food Front..

[50] Cervantes-Laurean D., Schramm D.D., Jacobson E.L., Halaweish I., Bruckner G.G., Boissonneault G.A. (2006). Inhibition of advanced glycation end product formation on collagen by rutin and its metabolites. J. Nutr. Biochem..

[51] Bardakci H., Celep E., Gözet T., Kurt-Celep I., Deniz I., Şen-Utsukarci B., Akaydin G. (2019). A comparative investigation on phenolic composition, antioxidant and antimicrobial potentials of *Salvia heldreichiana* Boiss. ex Bentham extracts. S. Afr. J. Bot..

[52] Celep E., Akyüz S., İnan Y., Yesilada E. (2018). Assessment of potential bioavailability of major phenolic compounds in *Lavandula stoechas* L. ssp. stoechas. Ind. Crops Prod..

[53] Celep E., Charehsaz M., Akyüz S., Acar E.T., Yesilada E. (2015). Effect of in vitro gastrointestinal digestion on the bioavailability of phenolic components and the antioxidant potentials of some Turkish fruit wines. Food Res. Int..

[54] Bardakci H., Celep E., Gözet T., Kan Y., Kırmızıbekmez H. (2019). Phytochemical characterization and antioxidant activities of the fruit extracts of several *Crataegus* taxa. S. Afr. J. Bot..

[55] Celep E., Aydın A., Yesilada E. (2012). A comparative study on the in vitro antioxidant potentials of three edible fruits: Cornelian cherry, Japanese persimmon and cherry laurel. Food Chem. Toxicol..

[56] Balan K., Ratha P., Prakash G., Viswanathamurthi P., Adisakwattana S., Palvannan T. (2017). Evaluation of in vitro α-amylase and α-glucosidase inhibitory potential of N_2_O_2_ schiff base Zn complex. Arab. J. Chem..

[57] Starowicz M., Zieliński H. (2019). Inhibition of advanced glycation end-product formation by high antioxidant-leveled spices commonly used in European cuisine. Antioxidants.

